# High-resolution and differential analysis of rat microglial markers in traumatic brain injury: conventional flow cytometric and bioinformatics analysis

**DOI:** 10.1038/s41598-020-68770-0

**Published:** 2020-07-20

**Authors:** Naama Toledano Furman, Assaf Gottlieb, Karthik S. Prabhakara, Supinder Bedi, Henry W. Caplan, Katherine A. Ruppert, Amit K. Srivastava, Scott D. Olson, Charles S. Cox

**Affiliations:** 1 Department of Pediatric Surgery, McGovern School of Medicine, University of Texas Health Science Center at Houston, 6431 Fannin St. MSB 5.230, Houston, TX 77030 USA; 2 Center for Precision Health, School of Biomedical Informatics, University of Texas Health Science Center, Houston, TX 77030 USA

**Keywords:** Microglia, Neuroimmunology, Experimental models of disease

## Abstract

Traumatic brain injury (TBI) results in a cascade of cellular responses, which produce neuroinflammation, partly due to microglial activation. Transforming from surveying to primed phenotypes, microglia undergo considerable molecular changes. However, specific microglial profiles in rat remain elusive due to tedious methodology and limited availability of reagents. Here, we present a flow cytometry-based analysis of rat microglia 24 h after TBI using the controlled cortical impact model, validated with a bioinformatics approach. Isolated microglia are analyzed for morphological changes and their expression of activation markers using flow cytometry, traditional gating-based analysis methods and support the data by employing bioinformatics statistical tools. We use CD45, CD11b/c, and p2y12 receptor to identify microglia and evaluate their activation state using CD32, CD86, RT1B, CD200R, and CD163. The results from logic-gated flow cytometry analysis was validated with bioinformatics-based analysis and machine learning algorithms to detect quantitative changes in morphology and marker expression in microglia due to activation following TBI.

## Introduction

Traumatic brain injury (TBI) is attributed to a third of all injury-related deaths in the United States^1^. Each year, 1.7 million people receive treatment for brain trauma, and more than 5 million people receive treatment for TBI-related complications and disabilities. TBI can occur in a variety of circumstances and a range of severity levels, which lead to cognitive and behavioral changes and increased risk for acquired neurodegenerative diseases (e.g., Alzheimer’s disease or Parkinson’s disease)^2–5^. Regardless of the severity of the damage caused to the brain tissue, a secondary sub-acute injury mechanism follows the initial injury involving the activation of resident microglia and infiltrating immune cells (such as neutrophils, monocytes, macrophages, lymphocytes etc.) that further contribute to neuroinflammation^6,7^. Similarities between microglia and macrophages complicate research, and may often result in considering macrophages and microglia a uniform unit that acts in response to inflammatory stimuli^8,9^. Methods for isolation of macrophages from non-CNS tissues are well established and as a result, macrophages are highly investigated. Despite sharing some functional activities with macrophages, it is not accurate to assume that microglia act in the same manner^10^.

**Figure 1:**
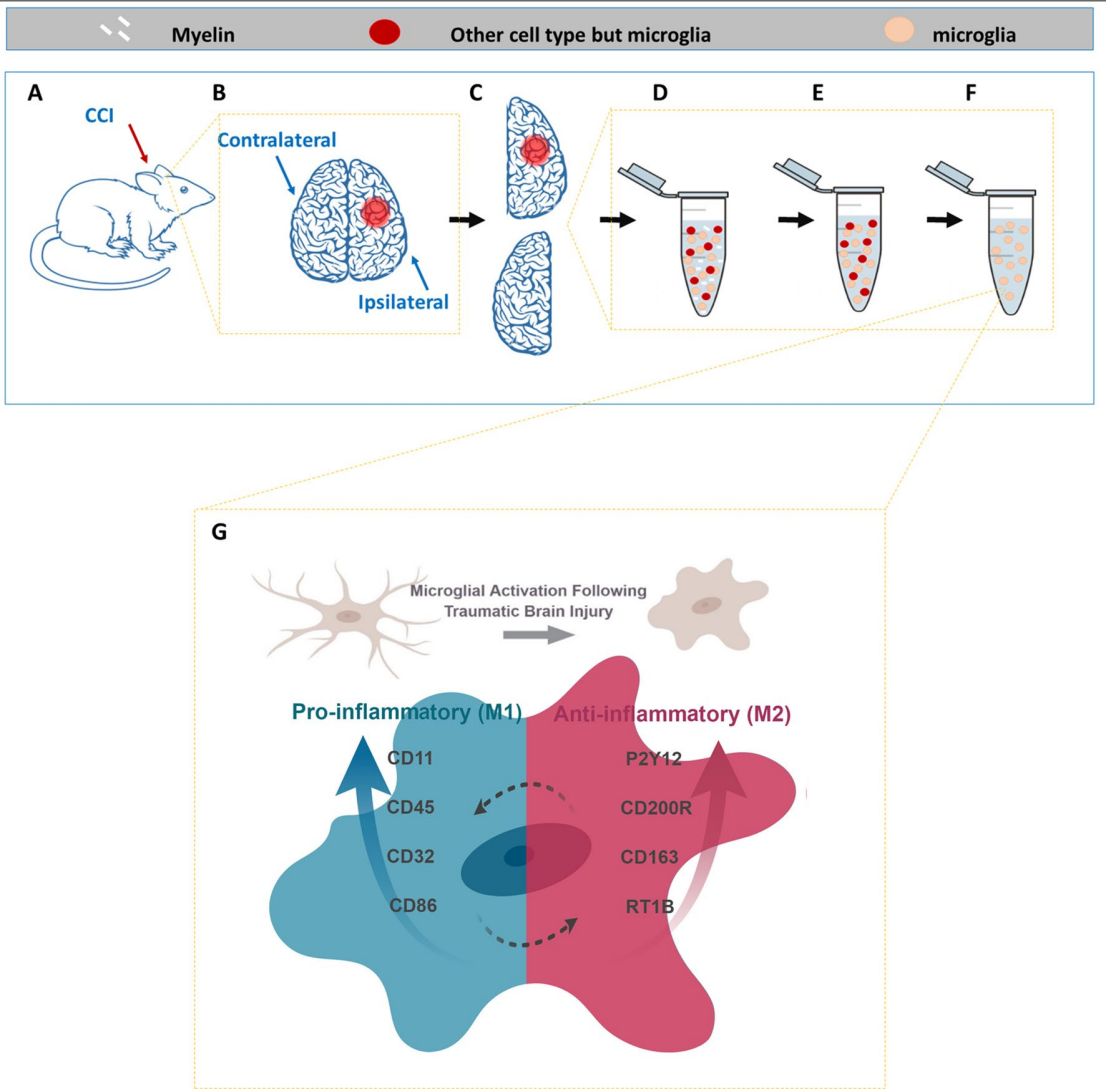
Process and Rationale Illustration. (**A**) Rats were injured using the controlled cortical impact method, to create a precise injury at one hemisphere. (**B**) Each hemisphere was processed to a separate sample, by mechanically and enzymatically digesting the tissue into single cell suspension (**D**), myelin removal (**E**) and microglial (CD11bc) enrichment (**F**), which is the final sample that was later analyzed with flow cytometry. (**G**) Considering the morphological changes and the changes in markers expression we used a validated multi-parametric flow cytometry panel to evaluate all parameters together to create a profile of the activated microglia at 24 after injury. Made in ©BioRender—biorender.com.

Microglia play a crucial role in both healthy and injured brains. They are constantly active and survey the brain for signals, often acting to remove cellular debris^11^. In normal conditions, microglia retain their ramified phenotype. Microglia activation occurs within minutes of injury or infection, resulting in a rapid change in gene expression. Activated microglia undergo dramatic morphological transformation^7^, which is required for their revised role in the neuroinflammatory response. Their newly acquired amoeboid shape makes them indistinguishable from the recruited blood derived macrophages responding damaged tissue^12^. Like macrophages, activated microglia can polarize into several phenotypic categories depending on the inflammatory stimuli, although current thinking allows partial or mixed activation states to create a more nuanced immune response, rather than rigid phenotypes^13^ (See graphical illustration, Fig. 1). The classical activation profile (M1) is in response to TNF-α and IFN-γ, and largely considered pro-inflammatory. The M1 pathway is associated with phagocytosis , ability to kill pathogens, and ROS release, in addition to other inflammatory cytokines in order to combat pathogens^14^. Since microglia are antigen presenting cells and communicate with T cells, activated M1 microglia upregulate their cell surface markers, such as MHC-II and CD86^15^.

The alternative, anti-inflammatory (M2) classification subdivides into three major alternative pathways. While the phenotypic profile of M2 microglia is diverse, they are uniform in their ability to downregulate inflammation, and protect or repair the CNS^16^. The first subdivision of M2 is when microglia are stimulated using IL4 or IL13 into an alternative pathway that includes immunity against parasites, Th2 cell recruitment, and tissue repair. These conditions define the cells as M2a-polarized. The second division is the M2c phenotype, also called “acquired deactivation”, which is in response to IL10, glucocorticoids, or uptake of apoptotic cells where microglia are involved in tissue remodeling processes. In M2c, cells will overexpress transforming growth factor beta (TGF-β), sphingosine kinase and CD163, the membrane-bound scavenger receptor for haptoglobin/hemoglobin complexes^17–19^. Lastly, the third division is the M2b phenotype which has characteristics of both M1 and M2, and is associated with memory immune response^20^.

Due to recent discoveries, we now know that microglia are a distinctive lineage and have an independent molecular signature that separates them from circulating monocytes and macrophages. Microglia and macrophages are different not only in their molecular signature and in their origin, but in the factors that regulate their development^10,21,22^. Recently revealed microglia-specific genes include P2y12, Fcrls, Tmem119, Offm113 and Tgfbr1. Assays based on those genes or gene products will precisely distinguish microglia from macrophages^7^. In this project, we use the purinergic receptor P2y12 to distinguish microglia from macrophages, as our options are constrained by the limited availability of anti-rat specific reagents. In addition to exclusive expression in microglia of the central nervous system, P2y12 receptors are involved in microglia membrane ruffling and chemotaxis^23^. Their overexpression contributes to process extensions, which is followed by migration toward injury^24^.

Although the two hemispheres of the brain can respond independently, they also coordinate for many functions. They can sense and signal each other and may even compensate over the compromised activity of the injured side, when needed^25^. We hypothesized that a focal cortical contusion in the brain would result in a phenotypic change in microglia and that the ipsilateral and contralateral hemispheres would develop their own unique phenotypic arrangement at the critical 24 h after injury, highlighting the differences between the neuroinflammatory responses in the injured microenvironment compared to the entire CNS. The purpose of this study was to provide high-resolution insight on both ipsilateral and contralateral microglial marker expression after injury using multi-color flow cytometry. Flow cytometry, in contrast to immunohistochemistry, is quantitative by default, fast, and can be adapted to assay numerous replicate samples with precision and reproducibility. Using an optimized protocol^26^ we recently developed in our lab, we were able to identify rat microglia and learn about their biological features, including morphology and surface marker expression by comparing ipsi- and contralateral hemispheres of sham injured and CCI injured rat brain tissues at 24 h after injury. The resulting data was analyzed with conventional flow cytometry techniques using logic gating strategies, as well as validating bioinformatics-based multi-parametric approaches that were not limited by assumptions or bias.

## Methods

### Animals

Male Sprague Dawley Rats (225–250 g, approximately 8wks old, Envigo Labs) were the source of CNS tissue. The usage of the animals was approved by the Animal welfare committee at University of Texas Health Science Center at Houston, Texas, protocol: AWC16-0046. Animals were handled in accordance with the standards of the American Association for the Accreditation of Laboratory Animal Care (AAALAC). Five-week old rats were housed in pairs under 12 h light/dark cycles in temperature-controlled conditions. Water and standard rodent laboratory chow were accessible ad libitum.

### Controlled cortical impact model

To establish TBI model in the rats, unilateral brain injury was induced using Impact One Stereotaxic Impactor (Leica Microsystems, Buffalo Grove, IL) a controlled cortical impact (CCI) device. Rats were anesthetized with 4% isoflurane/O_2_. After mounting their head in a stereotactic frame, they received a single impact to the right parietal association cortex of 1.0 mm depth of deformation with an impact velocity of 5.0 m/sec and a dwell time of 200 ms to achieve moderate to severe injury. The injury procedure was complete after the incision was closed with staples.

### Tissue dissociation and cell isolation

To apply flow cytometric methodology for cell characterization we dissociated the brain tissue into single cell suspension. Twenty-four hours after injury, rats were anesthetized with 4% isoflurane/ O_2_, and sacrificed via right atrial puncture. Brains were excised and the cerebellum was removed. Brains were divided to ipsilateral and contralateral hemispheres, for separate processing. The entire brain cell population was isolated from the brain tissue by enzymatic digestion and mechanical dissociation using Neural Tissue Dissociation Kit with GentleMACS (Miltenyi Biotec), according to manufacturer protocol with a few modifications to adjust the mouse neural tissue oriented protocol to accommodate rat brain tissue. Briefly, each brain hemisphere was placed in C tube and added with pre-warmed 4 ml of Buffer X and 100 µl of Enzyme P. After mechanical dissociation with the GentleMACS on program m_brain_01, cells were incubated on a tube rotator for 15 min at 37 °C. Following program m_brain_02, cells were added with 40 µl of Buffer Y and 20 µl of Enzyme A, and incubated for 10 min as before. For the last incubation, at the same conditions, we applied program m_brain_03. Cells were filtered using a 70 µm MACS strainer, centrifuged and washed once with HBSS (Gibco). Myelin removal was achieved by suspension of each brain hemisphere in 3 ml of 30% Percoll (GE Healthcare Life Sciences) in HBSS, placed in a 15 ml falcon tube. Cells were centrifuged at 700 g for 10 min, with no break, myelin accumulated at the top while cells were submerged into a pellet. After myelin aspiration, cells were washed in HBSS in a total volume of 12 ml to dilute the Percoll and centrifuged at 500× g for 10 min.

### Microglia enrichment

The cell pellet consisting of a mixture of all brain cells was further subjected to magnetic cells sorting for microglia enrichment using CD11b/c microbeads (Miltenyi Biotec), according to manufacturer protocol. The retrieved cells of the non-enriched and enriched fractions were suspended in microglia medium (ScienCell Research Labs. Inc.), and kept overnight until processing for flow cytometric analysis the next day.

### Flow cytometric multicolor panel

To specifically identify microglia and to characterize their pro- and anti-inflammatory phenotype, CD11b/c enriched cells were surface stained. The list of antibodies is summarized in our published optimized multicolor immune-phenotyping protocol^26^. Each sample of the CD11b/c enriched cells was five times diluted with staining buffer (Biolegend), washed and divided to M1 (pro-inflammatory path) and M2 (anti-inflammatory path) and added with the appropriate antibody mix. Cells were incubated for 30 min at RT in the dark. After another wash, cells were added with the secondary antibody mix and Ghost live/dead reagent and incubated for 20 min at RT in the dark. Cells were finally washed, added to counting control beads (Cyto-Cal™) and run. Data was acquired with LSRII cytometer with Diva acquisition software (BD Biociences, San Jose, CA). Analysis was conducted using Kaluza vr. 1.5a software (Backman Coulter, Brea, CA). Fluorescence spillover compensation values were generated using VersaComp Antibody Capture Beads (Beckman Coulter, Inc.). tSNE analysis was performed for each panel separately using FlowJo vr. 10, with down sampling and tSNE plugin that included all markers (but not FS and SS).

### Statistical analysis

Unless otherwise indicated, all values presented are mean ± SEM. Comparison between ipsilateral and contralateral hemispheres and CCI vs. sham were evaluated using Mann Whitney U test and Paired T Test. For other statistical analysis performed, see Bioinformatics analysis in the Results section. The bioinformatics analysis was performed in Matlab v9. For all experiments, a minimum of 10,000 cells were acquired from both the ipsilateral and contralateral hemisphere from three different animals (n = 3) per treatment group.

### Ethics approval and consent to participate

The usage of the animals was approved by the Animal welfare committee at University of Texas Health Science Center at Houston, Texas, protocol: AWC16-0046. Animals were handled in accordance with the standards of the American Association for the Accreditation of Laboratory Animal Care (AAALAC) (see Methods).

## Results

### Identification of microglia

**Figure 2:**
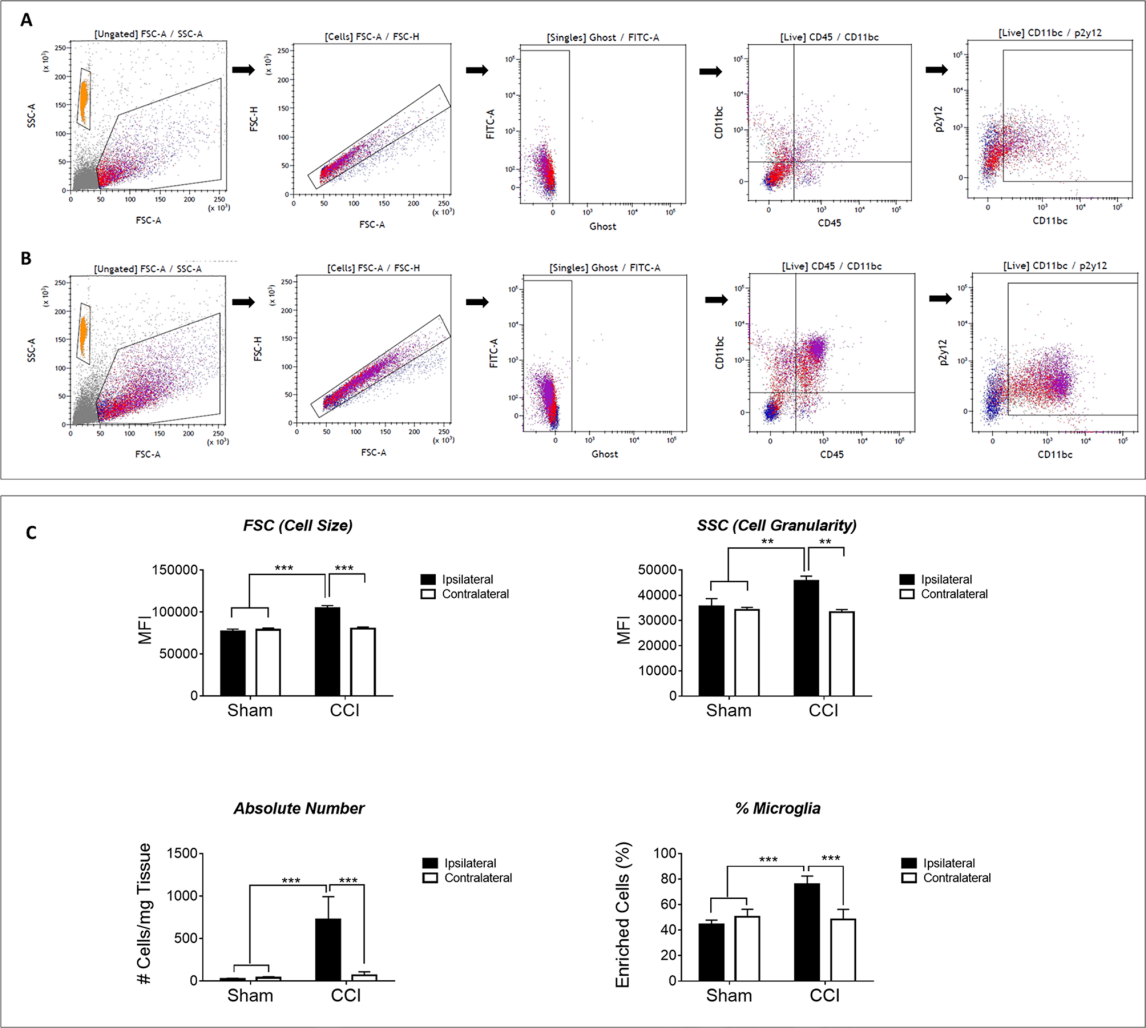
Gating Strategy and Microglia Identification at 24 h after Injury. Microglial cells were identified using the following gating strategy: cells were identified as the main population (with the orange population representing the counting beads), and then debris and cell aggregates were excluded using correlation of FSC area vs height. Dead cells were excluded using Ghost™ dye. For the visual comparison, microglia were identified as CD45^+^CD11b/c^+^P2y12^+^ cells. Panel **A** is representing sham ipsilateral while panel **B** represents CCI ipsilateral samples. Both are presented to imply on the dramatic changes that are observed only from this initial step. The enriched fraction of cells includes neutrophils, monocytes and macrophages that share similar markers to those of resident microglia (CD11bc but not only). Using P2y12 we were able to distinguish microglia (CD45^+^CD11b/c^+^P2y12^+^) from other cell types and characterize each subpopulation of the cells. (**C**) At 24 h after injury, we conclude, that the main population of cells underwent dramatic changes in size (FSC), granularity (SSC) and in its absolute cells number. We attribute these changes mostly to microglial cells as there are also increased in % from the total population observed. Values presented are mean values ± s.e.m (n = 3).

We developed our methodologies by analyzing the changes that occur in microglial populations of ipsilateral and contralateral brain hemispheres 24 h after injury (see illustration of the process in Fig. 1). First, enriched single-cell suspensions of brain-derived microglia were enumerated and assayed by flow cytometry for their physical properties- size and granularity. The main population of cells was identified through Forward Scattered light (FSC) and Side Scattered light (SSC). Generally, FSC is a measurement for the relative size of the event, while SSC is a measurement for the event granularity^27^. Single live cells were identified by exclusion of doublets and gating of viable cells using live/dead dye (Ghost Dye™). Microglia cells were identified as cells positive for CD45, CD11b/c and P2Y12 (Fig. 2).

### Microglial cell size and granularity following activation

Our sample analysis reveals a significant increase in the relative size of the microglia in the injury site (Wilcoxon rank sum test, *p* value ~ 0 (*p* value too small to report) between the ipsilateral of sham vs. injured groups, and between the ipsilateral and contralateral of the injured group. *p* value < e-^−22^ between contralateral of sham vs. injured groups) and in granularity (*p* value ~ 0 (too small to report)) between the ipsilateral of sham vs. injured groups, and between the ipsilateral and contralateral of the injured group on the injured side. *p* value = 4e^−5^ between contralateral of sham vs. injured groups) (Fig. 2C and Supp Table 1). For quantification, the cells were acquired with Cyto-Cal™ quantification beads and the absolute number of cells per mg of brain tissue was calculated. The ipsilateral hemispheres of the injured rat showed significantly increased percentage and number of cells per mg of tissue at 24 h after CCI compared to the other groups. The injured hemisphere contains 16 times more microglia relative to the average of the other groups (sham ipsilateral, sham contralateral and CCI contralateral) (Fig. 2C).

### Microglial change of marker expression profile following injury

**Figure 3:**
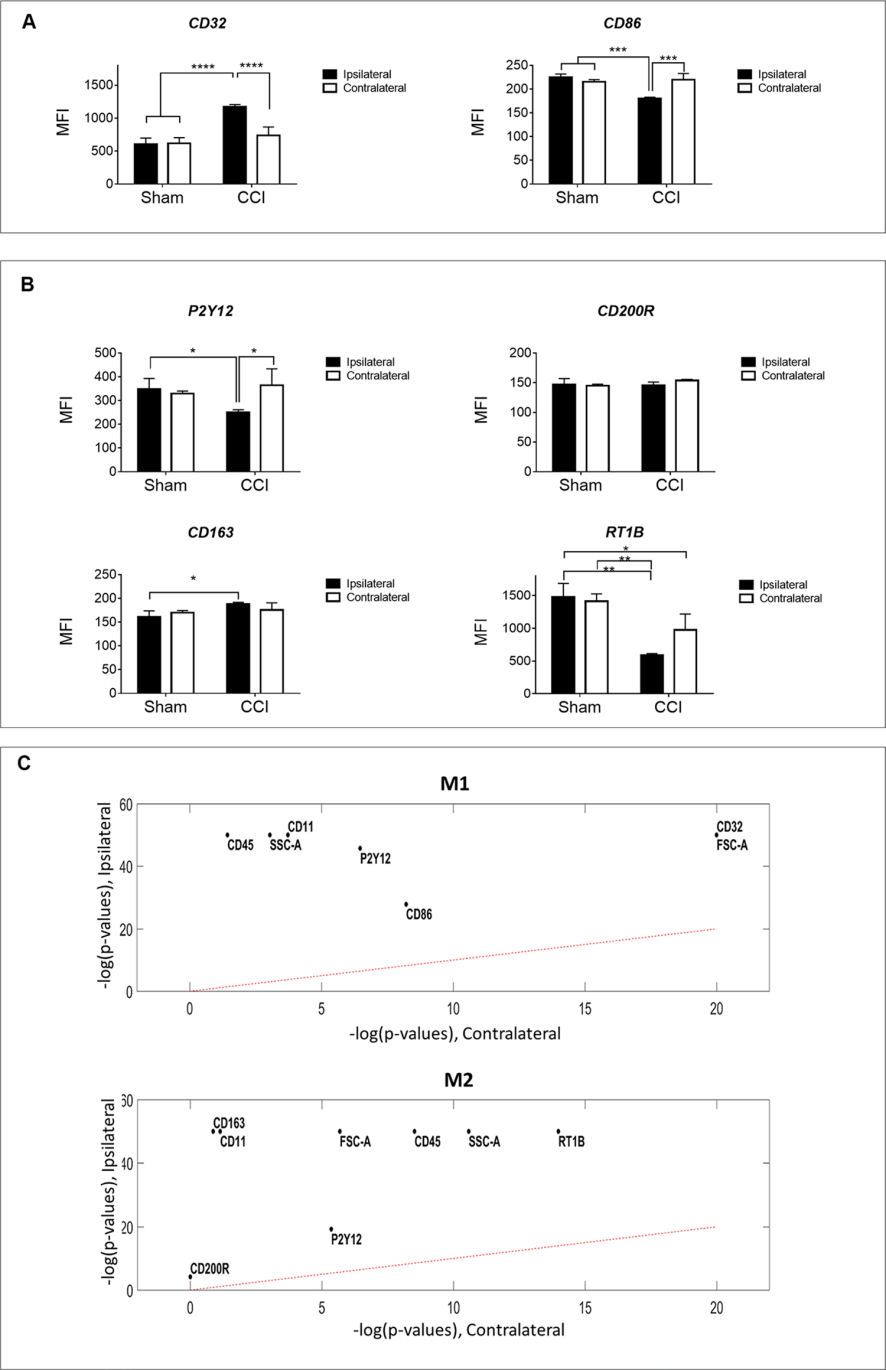
Microglia Polarization at 24 h after Injury (Traditional Analysis). Cells gated under CD45^+^CD11b/c^+^P2y12^+^ were further characterized according to M1 associated markers CD32 + or CD86 + (panel **A**) and M2 associated markers CD200R + ,RT1B + , or CD163 + (panel **B**). Mean fluorescence intensity (MFI) was measured in both ipsilateral and contralateral hemispheres of sham and CCI brains 24 h after injury. We conclude that these microglia subpopulations express significantly higher CD32 and CD163, while also expressing significantly lower CD86 and RT1B. Data represent mean values ± SD (n = 3). Statistical analysis performed by Two-Way ANOVA and Uncorrected Fisher’s Least Significant Difference (LSD) test. (**C**) Mann–Whitney U Test to evaluate the differences between the ipsilateral/contralateral event measurements and the corresponding sham measurements, adjusting for Benjamini–Hochberg false discovery rate (FDR) of 0.05.

While the percent of microglia (CD45^+^CD11^+^P2y12^+^) is significantly increased with injury (Fig. 2C), the measurement of the MFI of CD45, CD11b/c and p2y12 showed the following: the MFI of CD45 and CD11b/c were significantly increased in the injured hemisphere, while p2y12 MFI was significant decrease in (Fig. 3B). This suggests that at 24 h after injury, p2y12 presentation on microglia cell surface is inhibited.

To learn about the density/expression of a certain marker, we present their MFI values (Fig. 3). We focused our analysis on CD45, CD11b/c and P2y12 gated microglia, and profiled their phenotype with several characteristic markers for pro- and anti-inflammatory polarization. The two markers, potentially involved in the pro-inflammatory path, CD32 and CD86, presented an inconsistent trend at 24 h after injury. Ipsilateral hemispheres of CCI animals reveal CD32 MFI significantly increased following injury while CD86 MFI significantly decreased when compared to all other groups (Fig. 3A).

Elevation of expression in markers for CD200R, CD163, and RT1B is associated with the anti-inflammatory path. In the injured hemisphere, the expression of CD200R does not reflect a significant difference in MFI at 24 h after injury. For RT1B, we observed significantly lower MFI compared to all other groups. For CD163, the MFI value significantly increases compared to sham values. In summary, we concluded that at 24 h after injury, microglia have lower surface presentation of CD86 and RT1B (Fig. 3B).

### Bioinformatics analysis

To build a complete cell profile and validate the analysis, our traditional analysis is also supported by bioinformatics analysis and application of comprehensive statistical tests on the same data sets. The raw data (extracted from fcs files) was interrogated by applying the following statistical tests on the events gated as microglia cells by excluding debris, counting beads and non-microglial cells.

#### Single-marker analysis

**Figure 4:**
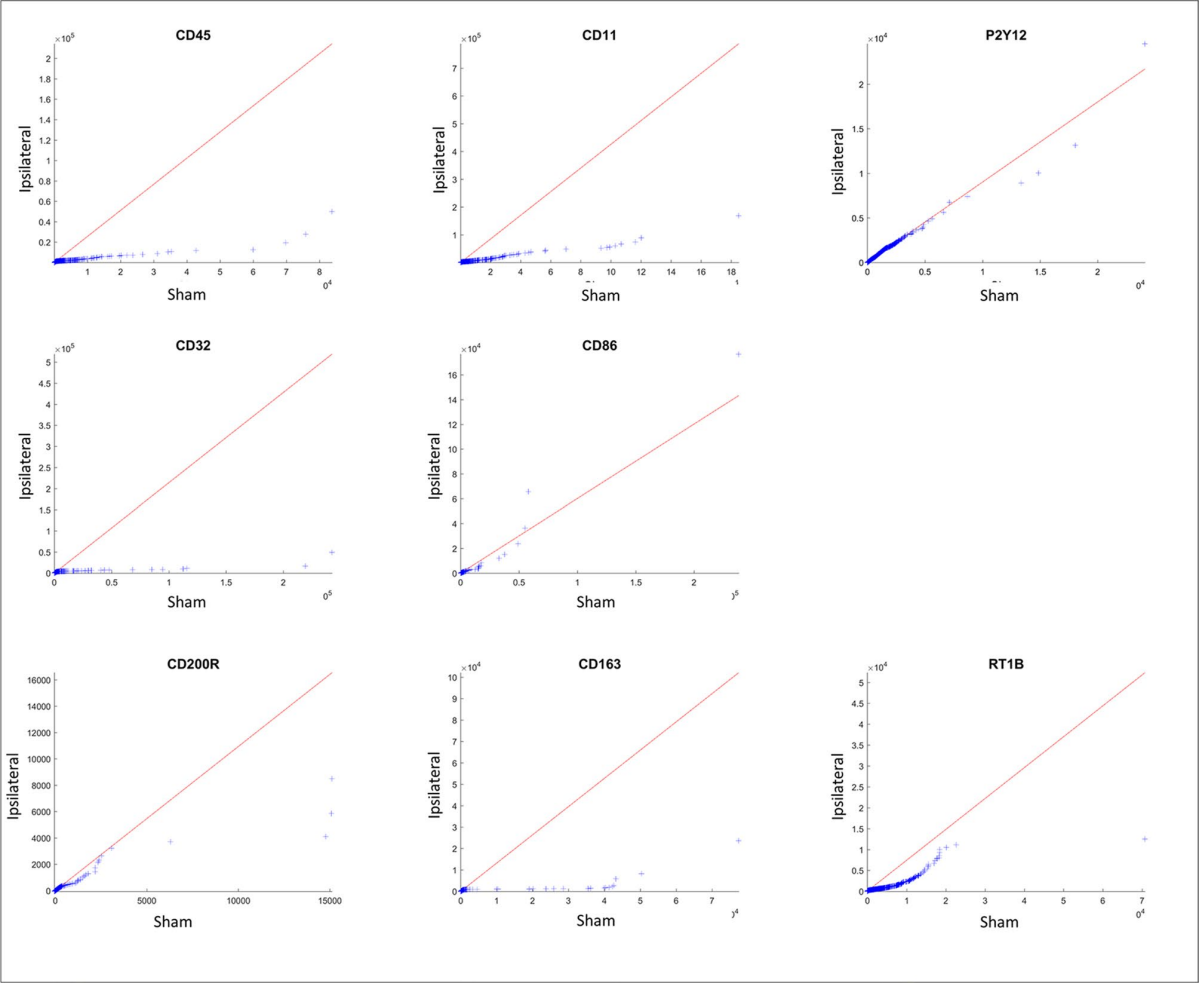
Changes in CCI Ipsilateral Marker Expression Profile Assessment with QQ (quantile quantile) Plots. A non-traditional method of looking at the flow data is to evaluate the change in distribution of each parameter tested, and presenting it with the probability plot, termed QQ plot. Ideally, when two populations share similar distribution, the points in the QQ plot will lie on the line y = x. In our case, the CCI ipsilateral data, when compared to the sham ipsilateral, is obviously skewed implying on a dramatic change in the marker expression.

We applied a Mann–Whitney U Test to evaluate the differences between the ipsilateral/contralateral event measurements and the corresponding sham measurements, adjusting for Benjamini- Hochberg false discovery rate (FDR) of 0.05 (Fig. 3C). In both ipsilateral and contralateral microglia, FSC and SSC are significantly different than sham. Out of the activation markers, all markers except CD200R are significantly different from sham in ipsilateral microglia and all markers except CD200R, CD163 and CD32 are significantly different from sham in contralateral microglia. The single factor analysis is also presented as QQ plot (Fig. 4, and Supplementary information Fig. 1) in which we present the parameters relative to their sham values. The QQ plot shows that the distribution of the activation markers in ipsilateral microglia CD32, CD11, CD163 and CD45 is markedly skewed towards to the left of the distribution relative to sham (more condensed), suggesting that these markers underwent dramatic changes as a result of the injury when compared to sham.

#### tSNE analysis

**Figure 5:**
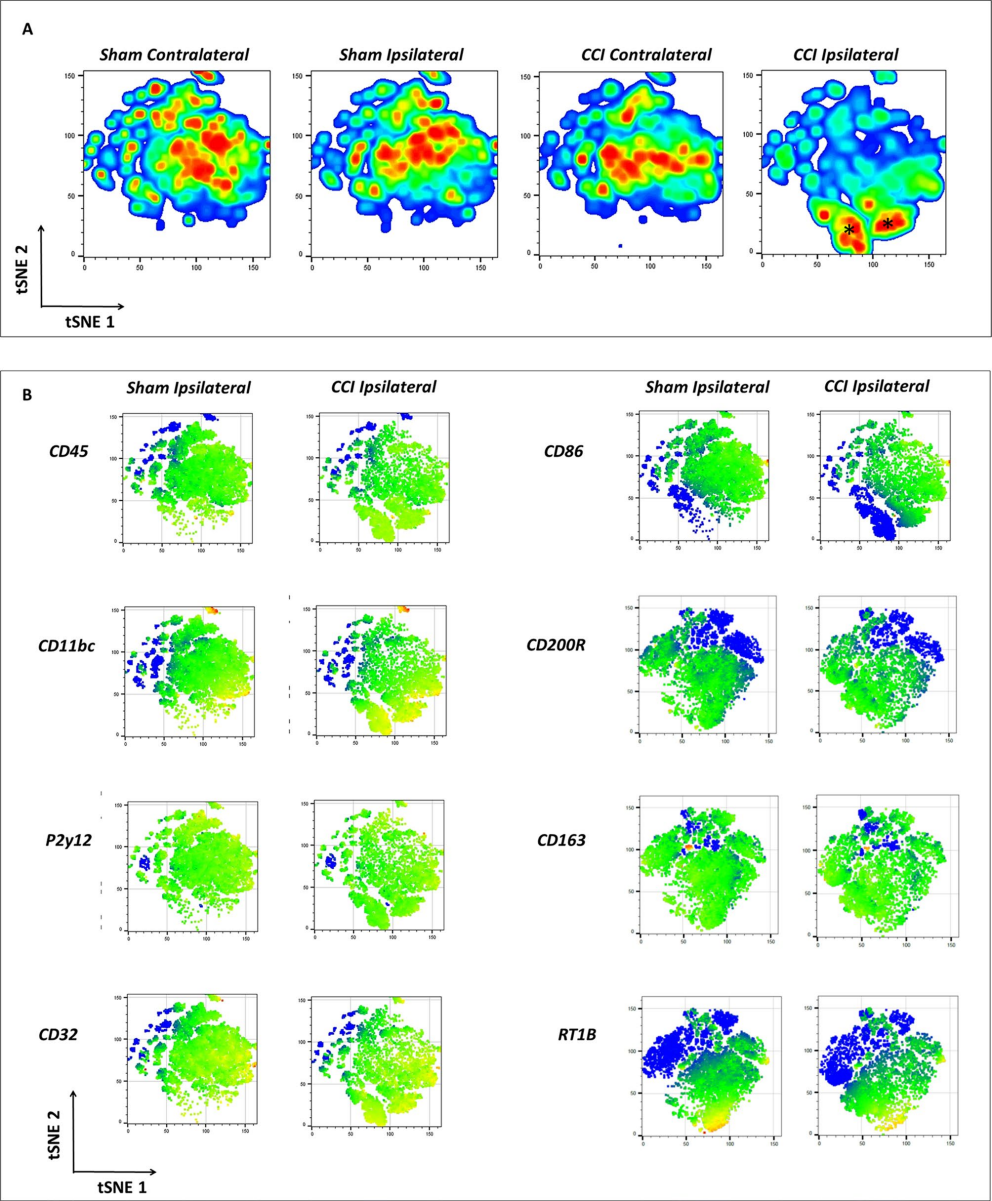
Assessment of Changes in Cell Density and Marker Expression following Injury Using tSNE. The machine-learning algorithm T-distributed Stochastic Neighbor Embedding (tSNE), which uses a nonlinear dimensionality reduction, was applied for visualization of the data. The islands created are determined by the algorithm contain events that share similar properties. The x- and y-axis and unites are determined by the algorithm (**A**) Presentation of total cell density changes comparing sham and injured with both contra-and ipsi-lateral hemispheres at 24 h after injury. The CCI ipsilateral shows a clear change with the formation of two distinct new islands. (**B**) Heat-maps of the mean values of the different markers used in the study are presented to visualize the changes the microglial population is undergoing at 24 h after injury. For comparison, the ipsilateral of sham and CCI are presented.

The t-distributed stochastic neighbor embedding (tSNE) data presentation allows the compression of the fluorescent parameters into two-dimension plot. However, this presentation need to be reported with caution as the assignment of groups gives arbitrary scale to each plot/group. Nonetheless, the obvious trends and principle of analyzing the data are clear. From the cell density presentation (Fig. 5A) we observe a similar pattern for sham contralateral and CCI contralateral groups. While sham ipsilateral mapping looks shifted a bit, the CCI ipsilateral looks completely different and presents new “islands” of cells (marked with *). These new islands are expressing microglial identification and activation markers, therefore, we assume they are composed of activated microglia. On these activated microglial cells, the tSNE analysis showed increased expression of CD45, CD11bc, but an overall decreased expression of P2Y12. The same pattern was observed when using tSNE for cluster mapping on the M2 panel (which includes CD45, CD11bc, P2y12 and the M2 associated markers CD200R, CD163 and RT1B).

As for the activation markers expression on the activated microglia: CD32 was shown to be highly expressed, CD86 is present but with low expression. As for the mean values of CD200R, RT1B and CD163 the tSNE presentation is not conclusive (Fig. 5B).

#### Relationships between markers

**Figure 6:**
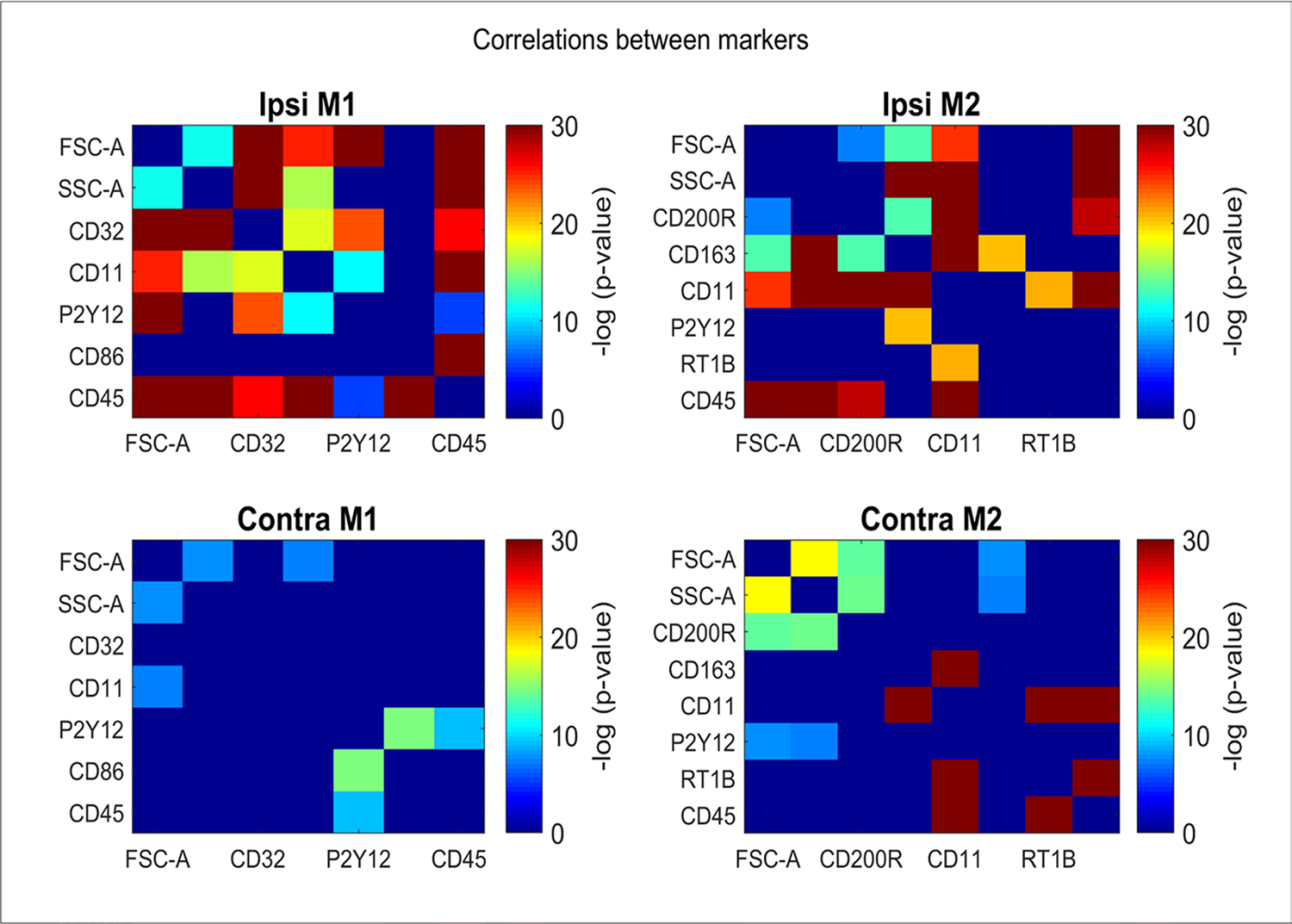
Assessment of Correlations between Markers. Heat maps presentation gathers the Cohen’s q [Cohen J. Statistical power analysis for the behavioral sciences] of the differences between the Pearson correlation coefficients between different activation markers across the registered events in the injured ipsilateral, contralateral relative to the same correlations in sham cells. Cold colors (green to blue) imply on non-correlative relationship between each couple of markers, while warm colors (yellow to red) represent increasing levels of correlation between each couple of markers. More correlations are observed in the ipsilateral compared to its contralateral hemisphere.

To evaluate relationships between the markers, we computed the Pearson correlation coefficients between different activation markers across the registered events in the injured ipsilateral and contralateral. We used Cohen’s q^28^ to estimate the significance of these correlations relative to correlations computed on the sham cells (Fig. 6). 56% of the ipsilateral injury correlations are significantly higher than their corresponding sham (Benjamini–Hochberg FDR of 0.01) while only 24% of the contralateral injury correlation are significantly different from their sham. Among these significant correlations, CD11 displayed high correlation with CD200R and with CD45 in the injured groups ipsilateral (ρ = 0.68 and ρ = 0.52, respectively, *p* ~ 0). Additionally, CD45 displayed high correlation with CD86 in the ipsilateral hemisphere (ρ = 0.58, *p* ~ 0). In contralateral injury, the only high and significantly different correlation than its corresponding sham was observed between CD11 and CD163 (ρ = 0.64, *p* ~ 0).

#### Markers differentiate between ipsilateral injury and sham

We tested the performance of a trained classifier to distinguish between injury and sham microglia and between injury ipsilateral and contralateral using the activation markers. We tested three state-of-the art machine learning classifiers, including logistic regression, random forest^29^ and radial basis function support vector machines in a tenfold cross validation scheme (averaged over 10 runs with different splits for robustness). The best performing classifier was the logistic regression classifier in discriminating between injury ipsilateral and sham (area under the receiver-operator curve (AUC) = 0.8 ± 0.0003 for M1 markers and AUC = 0.85 ± 0.00075 for M2 markers) and very similar performance in discriminating between ipsilateral and contralateral (AUC = 0.8 ± 0.001 for M1 markers and AUC = 0.83 ± 0.0002 for M2 markers). The predominant contributing activation markers to enable discrimination between ipsilateral injuries and either sham or contralateral were CD45, CD86 and RT1B. Interestingly, CD11 is a good discriminator between ipsilateral injury and sham, but less effective between ipsilateral and contralateral injuries. Supplementary Fig. 2 show the gradient difference between ipsilateral injury and sham using the logistic regression classifier score as propensity score of each microglia cell to deviate from sham. When applying the same classifiers to contralateral hemispheres, including both injured and sham groups, separation was close to random (best AUC obtained by random forest classifier = 0.55 ± 0.002).

## Discussion

In this study, we examine microglia to determine if a focal cortical contusion in the brain results in a phenotypic change in microglia, and whether the ipsilateral and contralateral hemispheres develop their own unique phenotypic arrangement 24 h after injury**.** Our goal is to provide high-resolution insight into both ipsilateral and contralateral microglial marker expression after injury using multi-color flow cytometry based data analysis. This method aims to distinguish local tissue CNS inflammatory events from global CNS neuroinflammation resulting from diffuse paracrine effects and systemic immune interactions.

We rapidly discriminate microglia from surrounding myeloid cells and profile their activation state and surface profile, indicating their role in ongoing neuroinflammation following TBI. To begin, we identify the single live cells, and then perform a general analysis of the cells as a group, see gating strategy in Fig. 2. We can barely discern changes in cell number or morphology 3 h after injury (data not shown). However, 24 h after injury, the number of cells occupying the ipsilateral hemisphere is higher compared to shams (Fig. 2), likely due to both cell infiltration and microgliosis. Infiltrating neutrophils and monocytes respond rapidly by crossing the blood–brain barrier and penetrating the injured neural tissue^1^. In addition, resident microglia, activate within minutes of injury^30^, proliferate and expand^31,32^.

Microglia morphology changes with activation to facilitate new biological functions^11^. These changes are readily visualized in immunohistochemistry imaging, previously performed by us and other groups^32–34^, demonstrating a transition from ramified resting microglia to an amoeboid morphology characteristic of a mobile, phagocytic cell. Kozlowski and Weimer^35^ developed an imaging-based, automated method to quantify microglia morphology. Their method allows the comparison of the mouse microglia for length, spread, roundness and soma size (cell body) before and after cell activation with LPS. With activation, cell length and spread decrease, while the roundness and soma size increase. In a recent study by Morrison et al*.*^36^, a similar strategy of using automated image processing and analysis methods were used to quantitatively describe rat microglial morphology following traumatic brain injury. A number of parameters were then used to statistically group microglia according to their membrane features, finding several distinct morphological identities that likely correspond to distinct activation states. In addition, Ritzel et al. correlates phagocytosis activity with granularity of microglia using flow cytometry^37^. These changes are detectable with flow cytometry through shifts in FSC and SSC to correlate predictably with the visual changes in cell shape and activity, despite mechanical and enzymatic isolation, selection, and assay as a single-cell suspension. As expected, our data analysis of the FSC and SSC parameters show the change in size and granularity (Fig. 2C). At this time point, the microglia in the ipsilateral hemisphere of the injured group is significantly larger and significantly more granulated when compared to the control groups.

Since the markers used to identify the cells are also changing with activation, we analyze the data for the median of fluorescent intensity (MFI) for each marker. In other words, it is reasonable to assume that all microglial cells express these markers at all times, while the level of expression of the markers changes according to the cell function. Moreover, a change in expression of a certain marker teaches us whether the cell direction is a pro- or anti-inflammatory path.

Microglia and macrophages respond to trauma in a dynamic manner. The literature implies that their initial phenotype is a transient anti-inflammatory (M2) phenotype, which switches within a week after injury to a sustained pro-inflammatory (M1) phenotype that can last for months and years^38–40^ . After identifying the microglial population (as CD45^+^CD11^+^P2y12^+^ cells), we analyze the cells for their activation marker profile. Since both pro- and anti-inflammatory paths associate with overexpression of the same markers, it is necessary to build a composite expression profile to better suggest/support one activation state over another. Still, additional parameters, such as cytokine expression and biological outcome are necessary. Due to microglial heterogeneity and the different roles microglia can acquire with activation^11^, we assume that the microglial reaction to different stimuli would result in a unique expression profile, which changes over time or with treatment, and may vary between patients (depending on gender, age, severity of injury etc.). Other acute CNS injury models, such as spinal cord injury and ischemic brain injury, also report mixed pro- and anti-inflammatory profile expression by activated microglia and macrophages^39,40^.

Recent investigations of the P2y12 receptor focus on its expression, role, and function in microglial activation^41^. The P2y12 receptor participates in acute inflammation responses^42^. Tatsumi et al., state that blockage of the receptor prevents microglial activation in a spinal microglia neuropathic pain model^42^. Moore et al., demonstrate in vitro that the expression of P2y12 increases on microglia during anti-inflammatory conditions (M2), compared to surveying microglia or microglia activated to a pro-inflammatory phenotype (M1) in both human fetal and adult microglia^24^. The differential analysis we perform at 24 h after injury suggests that p2y12 MFI is decreased with injury in ipsilateral (*p* < 0.007, Supplementary information Table 1) but is similar in contralateral between injury and sham. In agreement with our results, Hernandez et al., show a significant 20% reduction in P2y12 expression following brain injury in a mouse model^43^.

The markers CD32, CD86, CD163 and RT1B (Fig. 3, Supplementary information Table 1) show clear and significant changes. At 24 h after injury, activated microglia have a significant decrease in CD86 expression in the ipsilateral hemispheres of the injured group. We see the same trend in RT1B expression. Based upon the literature, CD86 and RT1B share common overexpression on M1, the pro-inflammatory path^44^. M2 activated microglia overexpress both, as well. Comparing our results with other publications, we learn that CD86 expression 24 h after injury is low and peaks 5 days post injury^8^ or later at 28 days post injury^45^. Direct comparison to other studies is difficult due to a lack of publications that describe rat microglial RT1B expression in acute CNS injury in vivo models. Functionally, antigen presenting cells express both CD86 and RT1B and help mediate microglial communication with T lymphocytes^11^. Since T cell infiltration occurs 5–7 days post injury^1^, the expression of these markers may not increase until later stages of injury.

As long as the microglial CD200 receptor binds its neuronal ligand CD200, the microglia do not activate^46,47^. In vitro studies show that CD200R expression is associated with microglia activation on the M2a path^47^. However, to the best of our knowledge, there is no confirmation of CD200R expression in the rat CCI model in vivo. Here, we show that the expression levels of CD200R stay constant at 24 h from injury (Fig. 3, Supplementary information Table 1).

In rat TBI models, we know very little about the expression of CD32. CD32, classically a pro-inflammatory (M1) marker, participates in inflammation regulation and phagocytosis^48^. The expression level of CD32 is shown to increase in both mRNA and protein levels 24 h after TBI in a mouse model, with peak expression at day 5^38^. In our case, TBI rat model at 24 h, CD32 levels were significantly increased in the ipsilateral of the injured group, compared to all other groups. CD163 is an anti-inflammatory phagocytic marker that associates with the M2c path. Until recently, CD163 expression was thought to be limited to macrophages^49,50^, but it has now been established that activated microglia express it as well^17,51^. There is a link between upregulation of CD163 and shorter disease course in Parkinson disease, while its overexpression results in a decrease in inflammation^17,52^. Our results show that both CD32 and CD163 are increased in the ipsilateral of the injured group compared to sham. Zhang et al., describe an accumulation of CD163^+^ cells at the lesion within 48 h in a rat TBI model^53^. However, this study does not discriminate between activated macrophages and microglia. Similar to our results, Turtzo et al., examine CD163 expression on the RNA and protein levels in macrophage and microglia in a rat model of CCI, finding initial low levels of both RNA and protein expression, which gradually increase and peak at 5 days after injury^8^. Our method is capable of improving on these studies by specifically profiling microglia separate from infiltrating myeloid cells.

Using bioinformatics, we validate our “traditional” flow cytometry analysis. We chose to apply the tSNE algorithm in order to obtain an additional unbiased way of analyzing our data. For the most part, it reassured our traditional results and helped us determine the isolated microglial profile at this time point. The cell density plots made it clear of how dramatic is the change the cell population is undergoing with injury. While most changes in the markers MFI were obvious and reassuring (Fig. 5), they needs to be reported with caution, and conclusions still need to be fully supported by quantitative analysis.

Next, we observe that multiple markers become highly correlated in 24 h in ipsilateral injury relative to sham, while much less so in contralateral injury, involving markers such as CD11, CD200R and CD45 (Fig. 6). Logistic regression classifier reveals an AUC of 0.85 based the M2 path active markers and AUC = 0.8 based on the M1 path when differentiating between ipsilateral hemispheres of injured to sham groups (Supp Table 2). However, this difference is absent for the comparison of the contralateral hemispheres of these two groups. These observations strengthen our conclusion that injury results in a unique molecular profile that is predictable at a given time point. This tool is very important, not only for validating our ability to properly and objectively evaluate the samples in this model, but also to serve as a predicting tool to determine efficacy of given treatment using the molecular signature of the microglia over a time course.

As we have shown, this data set is has a number of strengths and can be used for additional analysis, however, there are notable limitations to this technique, particularly compared to direct imaging techniques like immunohistochemistry. Flow cytometry can rapidly quantify surface markers on thousands of microglia, but it does not specify the origin of the cells, making it difficult to connect microglial phenotypes to specific brain regions without additional experiments. Similarly, while forward scatter and side scatter measurements are related to cell morphology, they are unable to conclusively identify whether microglia may be ramified, ameboid, rod-shaped or any other distinct shape associated with activation state^36^. Both a strength and a weakness, flow cytometry is most often limited to identifying and measuring surface epitopes. On one hand, this limits analysis from using intracellular antibodies to identify cells using unique markers, like transcription factors or mitochondrial proteins. On the other hand, flow cytometry can measure the amount of a marker present on the cell surface, which is particularly useful when a cell may use intracellular stores to rapidly change activation or phenotype using existing intracellular stores. While the amount of time and effort required to perform quantitative IHC was prohibitive, it was our growing appreciation for a continuous spectrum of microglial activation and polarization that created the need to resolve rapid and complicated changes in microglial phenotypes and this study.

Emerging technology and recent publications have demonstrated the usefulness of other quantitative methods that can identify microglial phenotypic changes in humans with high throughput, such as single cell RNAseq and mass cytometry using CyTOF^54-56^. RNA-based analysis does not account for multiple layers of post-transcriptional regulation that are active in microglia. Mass cytometry-based analysis is limited by reagent availability, as well as most the same limitations of our own study. These advanced techniques are most limited by their technical challenges and expense compared to the relative ubiquity and accessibility of multicolor flow cytometry. It is certain that a combination of techniques and assays is required to fully understand the dynamic nature of microglia in response to injury.

## Conclusion

We are able to use multi-parametric flow cytometry with a combination of high-resolution morphological gating, microglia specific markers, and unbiased statistical analysis to profile the microglial cells underlying neuroinflammation following injury, including the amount and level of activation among surveying versus reactive microglia and an accurate profile of the cells at 24 h after injury. This method is capable of assessing microglia, regardless of the time point, source or model. We can now see in high-resolution the differences between sham and CCI microglia, and even compare microglia between the contra- and ipsilateral hemispheres of the same group. These results confirm that the ipsilateral hemisphere is most affected by injury. Using a set of activation markers, we found significant differences between contra- and ipsilateral hemisphere microglial activation and phenotype. Unsurprisingly, determining pro- or anti-inflammatory designation of activated microglia is complicated and requires additional future studies. Our application of multicolor flow cytometry is potentially very useful, as changes in the activation marker expression profile in microglia indicate a change of function, immunological activity, and may be useful to measure the efficacy of an immunomodulatory therapy.

## Supplementary information

Supplementary information 1.

Supplementary information 2.

## Data Availability

The datasets during and/or analyzed during the current study available from the corresponding author on reasonable request and uploaded as supplemental file.
