# Supplementary material for: High-resolution and differential analysis of rat microglial markers in traumatic brain injury: conventional flow cytometric and bioinformatics analysis
Source: Sci Rep. 2020 Jul 20;10:11991. doi: 10.1038/s41598-020-68770-0 (PMC7371644; doi:10.1038/s41598-020-68770-0)

High-Resolution and Differential Analysis of Rat Microglial Markers in Traumatic Brain Injury: Conventional Flow Cytometric and Bioinformatics Analysis

Naama Toledano-Furman[1], Assaf Gottlieb[2], Karthik S. Prabhakara[1], Supinder Bedi[1], Henry W. Caplan[1], Katherine A. Ruppert[1], Amit K. Srivastava[1], *Scott D. Olson[1], Charles S. Cox, Jr.[1]

[1] Department of Pediatric Surgery, McGovern School of Medicine, University of Texas Health Science Center at Houston, 6431 Fannin St. MSB 5.230, Houston, Texas, 77030.

[2] Center for Precision Health, School of Biomedical Informatics, University of Texas Health Science Center, Houston, Texas 77030.

* Correspondence: Scott D. Olson Scott.D.Olson@uth.tmc.edu

Supplementary Figure Legend

Supplementary Figure 1: Changes in CCI Contralateral Marker Expression Profile Assessment with QQ (quantile quantile) Plots. A non-traditional method of looking at the flow data is to evaluate the change in distribution of each parameter tested, and presenting it with the probability plot, termed QQ plot. Ideally, when two populations share similar distribution, the points in the QQ plot will lie on the line y = x. In our case, the CCI contralateral data, when compared to the counter sham, is skewed which implies on a dramatic change in the marker expression.

Supplementary Figure 2: Markers Differentiate Between Ipsilateral Injury and Sham. Logistic regression classifier was applied to differentiate between injury ipsilateral and sham. Area under the receiver-operator curve (AUC) = 0.8±0.0003 for M1 markers and AUC=0.85±0.00075 for M2 markers. The predominant contributing features to differentiate between ipsilateral injuries and either sham or contralateral were CD45 and CD200R. The observed gradient difference between ipsilateral injury and sham using the logistic regression classifier score as propensity score of each microglia cell to deviate from sham. When applying the same classifiers to contralateral hemispheres, including both injured and sham groups, separation was close to random (best AUC obtained by random forest = 0.55±0.002).

Supplementary File: NTF 24hr TBI data.zip The Matlab workspace contains the following variables:

corrs - a structure containing correlations between the 13 measured covariates for

- ipsiM1 - Ipsilateral M1
- ipsiM2 - Ipsilateral M2
- contraM1 - Contralateral M1
- contraM2 - Contralateral M2
- sham_ipsiM1 - Sham Ipsilateral M1
- sham_ipsiM2 - Sham Ipsilateral M2
- sham_contraM1 - Sham Contralateral M1
- sham_contraM2 - Sham Contralateral M2

data - a structure containing the gated data with 13 measured covariates for

- ipsiM1 - Ipsilateral M1
- ipsiM2 - Ipsilateral M2

contraM1 - Contralateral M1

contraM2 - Contralateral M2

sham_ipsiM1 - Sham Ipsilateral M1

sham_ipsiM2 - Sham Ipsilateral M2

sham_contraM1 - Sham Contralateral M1

sham_contraM2 - Sham Contralateral M2

featureNames - a structure containing the name of the measured covariates for M1 and M2

relevantFeatures - a structure containing the indexes of covariates used for classification for M1 and M2

stats - a structure containing the AUC values for

10-fold cross validation (with 10 runs, each with different splits)

for Ipsilateral and Contralateral relative to their respective Shams

LR - logistic regression

RF - Random Forest

SVM - Support Vector Machine

weights - the weights of the logistic regression classifier for each covariate

Only Ipsilateral contains weights due to low performance of Contralateral

The weights are calculated across 10 different runs of 10-fold cross validation

Supplementary material

Supplementary Table 1: Mann Whitney U test results comparison between CCI and Sham.

Grey cells contain values that did not pass false discovery rate of 0.01.

	Ipsilateral			Contralateral		
**Marker**	**P-value**	**Ipsilateral values (Mean ± Stdev)**	**Sham values (Mean ± Stdev)**	**P-value**	**Contralateral values (Mean ± Stdev)**	**Sham values (Mean ± Stdev)**
FSC-A	0	112585±32851	87922±27221	E^{-22}	92355±30279	88641±28028
FSC-H	0	80889±22577	62422±18770	$3E^{-27}$	65642±20543	62922±19312
FSC-W	$2E^{-22}$	91254±6988	92663±8671	0.3	92324±8001	92641±8569
SSC-A	0	56896±28330	43444±19262	$4E^{-5}$	41850±20589	42233±19383
CD32	$2E^{-31}$6	1305±1955	1101±6209	$7E^{-39}$	1169±5526	1018±5223
CD11	0	1605±3421	937±4496	0.2	943±3887	934±5004
P2Y12	0.007	469±518	488±564	0.4	513±511	493±447
CD86	$8E^{-13}$	241±3057	347±3853	$3E^{-4}$	276±1845	324±2848
CD45	0	862±892	538±2054	$3E^{-4}$	499±2109	506±1834
CD200R	0.01	82±222	89±451	1	79±178	96±599
CD163	$3E^{-44}$	198±440	309±2516	0.4	269±3078	258±1806
RT1B	$2E^{-105}$	295±670	793±2403	$8E^{-7}$	516±4335	820±4304

Supplementary Table 2: Classification performance for separating CCI from Sham (AUC)

	1 day			
**Classifier name**	**Ipsilateral, M1**	**Ipsilateral, M2**	**Contralateral, M1**	**Contralateral, M2**
Logistic Regression	0.8±0.0002	0.85±0.0004	0.52±0.0005	0.53±0.002
Random Forrest (100 Trees)	0.78±0.002	0.78±0.001	0.55±0.002	0.54±0.005
Radial Basis Function Support Vector Machines	0.75±0.002	0.76±0.004	0.52±0.001	0.53±0.003

Supplementary Figure 1

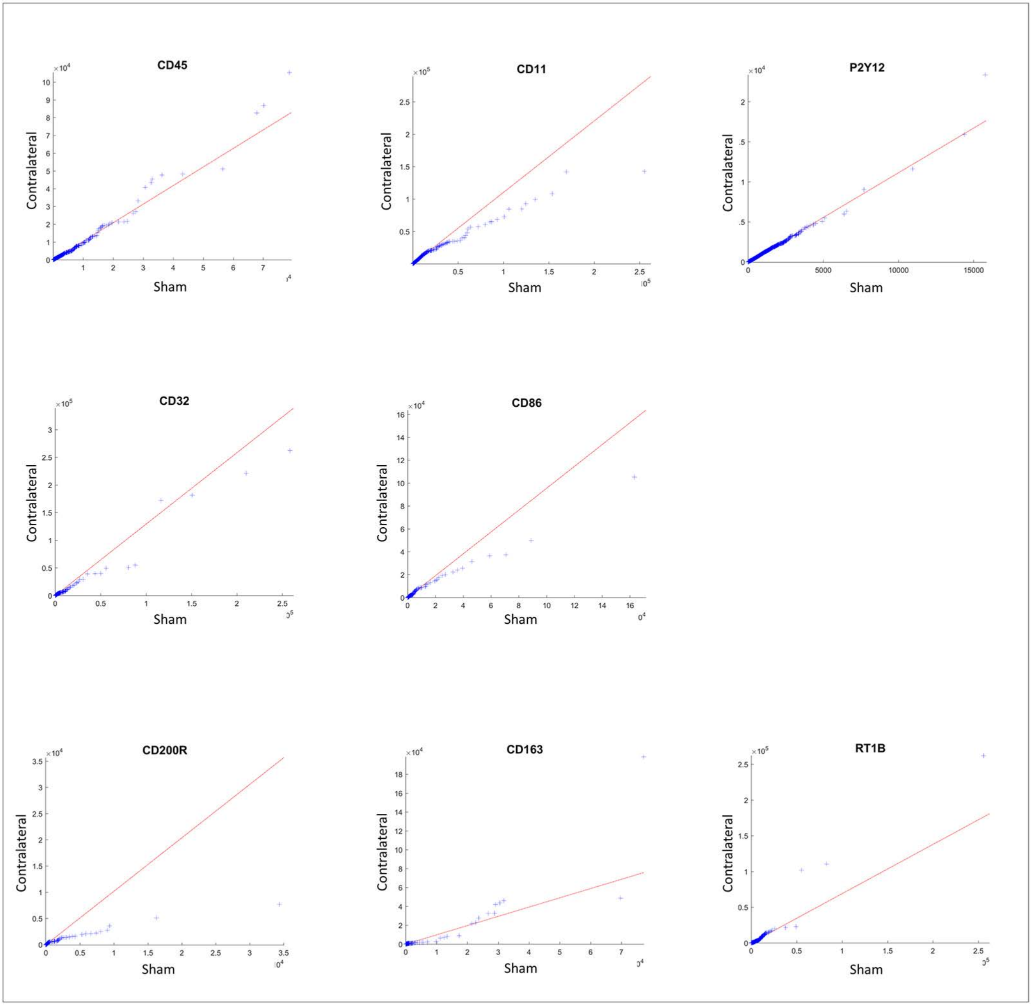

Supplementary Figure 2

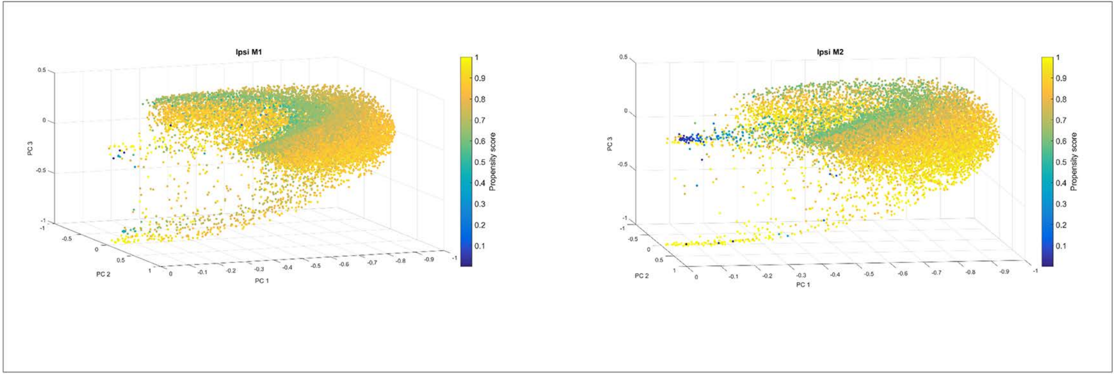

Supplement: Supplementary file 1 — Supplementary information 1. [file 41598_2020_68770_MOESM1_ESM.pdf]
